# Discovery of a mud‐covering cephalopod evidences the complex life habits in the abyss

**DOI:** 10.1002/ecy.70257

**Published:** 2025-11-25

**Authors:** Alejandra Mejía‐Saenz, Bethany F. M. Fleming, Daniel O. B. Jones, Loïc Van Audenhaege, Henk‐Jan Hoving, Erik Simon‐Lledó

**Affiliations:** ^1^ Scottish Association for Marine Science University of the Highlands and Islands Oban UK; ^2^ Ocean BioGeosciences National Oceanography Centre Southampton UK; ^3^ Ocean and Earth Science University of Southampton Southampton UK; ^4^ GEOMAR, Helmholtz Centre for Ocean Research Kiel Kiel Germany; ^5^ Institut de Ciències del Mar, ICM‐CSIC Barcelona Spain

**Keywords:** abyssal plain, aggressive mimicry, animal behavior, Cephalopoda, Clarion‐Clipperton Zone, Mastigoteuthidae

Cephalopods, including octopuses and squids, are a conspicuous component of marine ecosystems, present across all ocean depths (Jereb & Roper, 2010). At least 42 of the 50 described cephalopod families occur in the deep ocean, including the charismatic giant (*Architeuthis* spp.) and colossal squids (*Mesonychoteuthis hamiltoni*) in the midwater and cirrate octopods in the benthic boundary layer (Hoving et al., 2014). However, little is known about the distribution, diversity, and life habits of deep‐sea cephalopods, especially on abyssal plains (3000–6000 m depth), among the least studied features of our planet despite comprising over half its surface area. The seemingly low species abundance there, combined with avoidance behaviors, makes abyssal cephalopods elusive and difficult to study. In March 2023, at 4100 m depth, we captured unexpected mud‐covering behavior of an undescribed species of whiplash squid (Figure 1) within the Clarion‐Clipperton Zone (CCZ), an area in the abyssal central Pacific targeted for seabed mining. The squid was partially covered in mud with vertically exposed, rigid tentacles appearing to imitate biogenic stalks. This finding adds to the evidence of complex life habits in abyssal cephalopods (Golikov et al., 2023; Purser et al., 2016) and suggests seafloor sedimentary structure should be considered in understanding their distribution.

**FIGURE 1:**
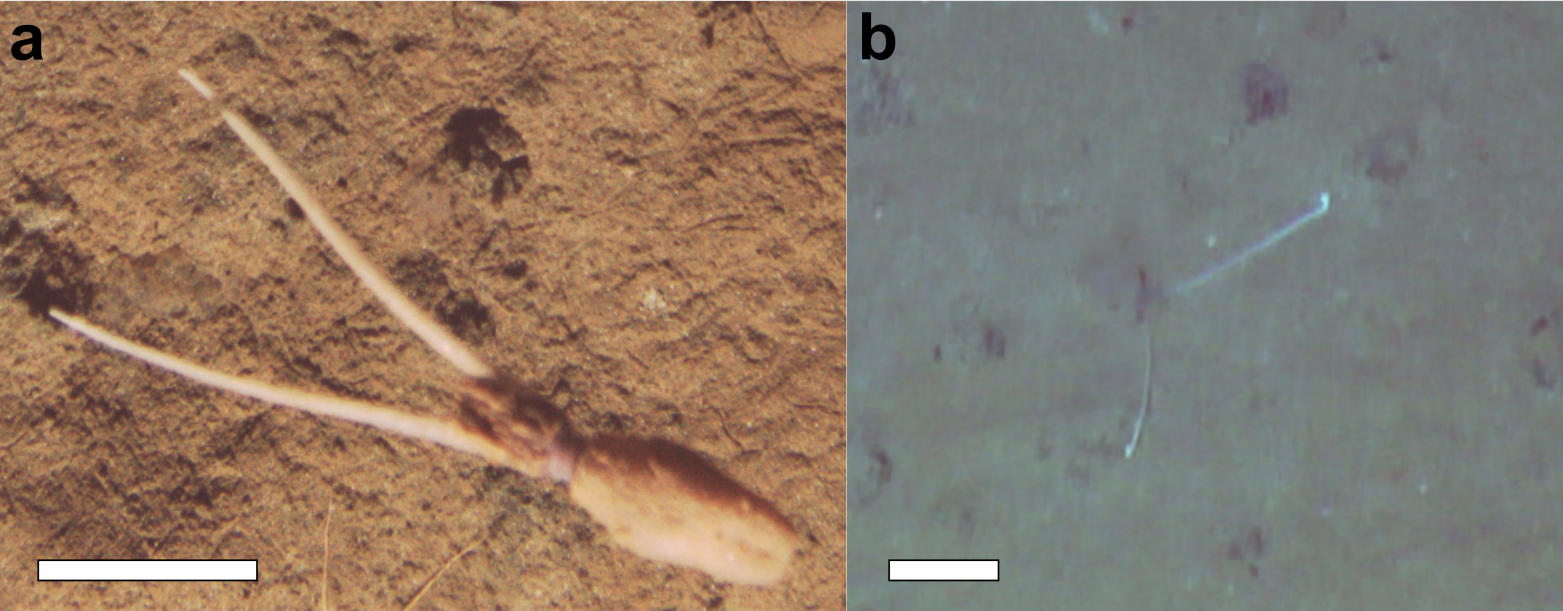
Behavior observed of a specimen of whiplash squid Mastigoteuthidae gen. indet. using the remotely operated vehicle (ROV) *Isis* during a seabed survey conducted at 4100 m depth in the abyssal Northeast Pacific (Clarion‐Clipperton Zone) on 17 March 2023. (a) Specimen swimming perpendicular to the seafloor, recorded with downward‐facing camera; (b) Specimen covered in soft sediment, motionless, with tentacles extended toward the water column, recorded in oblique‐facing footage ~4 s before image “a.” Scale bars: 10 cm. Full sequence: Appendix S1: Videos S1 and S2. Image credit: National Oceanography Centre/SMARTEX Project (NERC).

We were conducting visual surveys of the abyssal seafloor in the eastern CCZ (UK‐1 exploration area; position 13°58′1**″** N 116°32′34**″** W) using a remotely operated vehicle (ROV), an underwater robot, equipped with multiple cameras (Appendix S1: Figure S1; Jones & Glover, 2023). During one of the transects, systematic video recordings of the seafloor as the ROV moved forward, we captured a whiplash squid passing directly beneath the ROV and entering the field of view of the downward‐facing camera (Figure 1a; Appendix S1: Figure S2, Video S1). This squid morphotype (i.e., potential species) had been spotted only once before in more than 40 years of seabed exploration in the region (Simon‐Lledó et al., 2023b, see below) and could not be matched to any described species. Owing to its size (mantle length: ~10 cm; tentacle length: ~22 cm), wide elliptical fins, relatively short arms, and long bright white tentacles, we believe it belongs to the family of Mastigoteuthidae. Intriguingly, the specimen was not initially detected in the footage from the forward‐ nor oblique‐facing cameras, which captured the seconds just before it came into view below the vehicle. However, on closer inspection, we discovered that the squid had been buried under mud, between polymetallic nodules, and only emerged moments before being recorded in the water column (Figure 1b; Appendix S1: Video S2). In the angled video, it can be seen motionless, positioned upside‐down with the siphon and both of its elongated tentacles extending rigidly toward the water column. The squid only became obvious after it abandoned this behavior and swam away, disturbed by the proximity of the ROV. This was puzzling as mastigoteuthids, including the only other specimen detected at the CCZ, typically occur near the bottom in a tuning fork posture (Roper & Vecchione, 1997; Figure 2b). To our knowledge, this is the first time that upside‐down burying is documented for squid. We hypothesize that this behavior constitutes a camouflage that combines masquerade and aggressive mimicry as biological substrate, which is also novel for this charismatic taxon.

**FIGURE 2:**
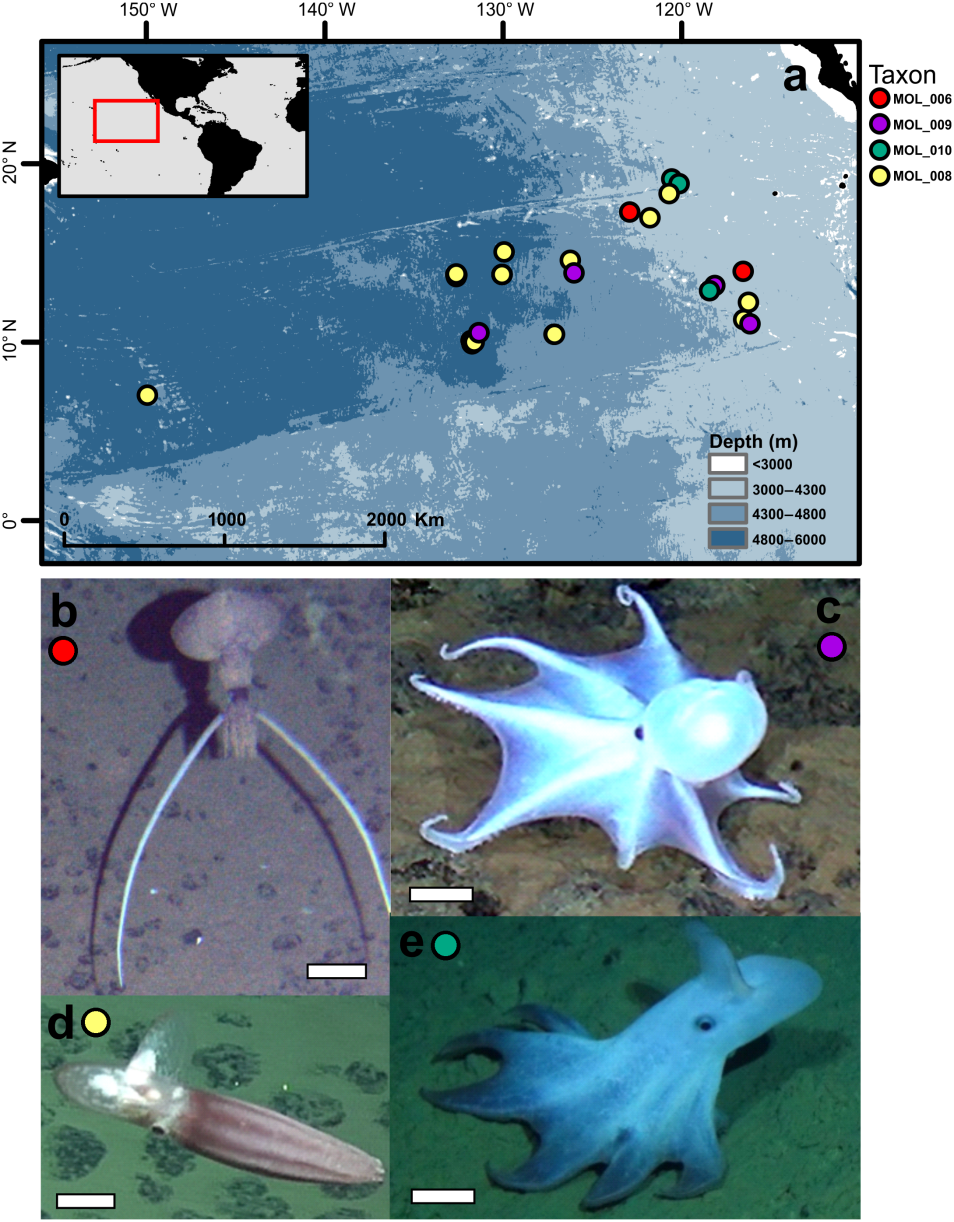
Cephalopod occurrences at the abyssal Northeast Pacific. (a) Map showing all the sightings per taxon. (b) Whiplash squids Mastigoteuthidae gen. indet. (MOL_006). (c) “Casper” octopus Octopodidae gen. indet. (MOL_009). (d) Big‐finned jellyhead octopus *Cirroteuthis muelleri* sp. inc. (MOL_008). (e) “Dumbo” octopus *Grimpoteuthis* sp. indet. (MOL_010). Scale bars: 5 cm. Nomenclature and animal image source: Abyssal Pacific Megafauna Atlas (Simon‐Lledó et al., 2023c in Zenodo at https://doi.org/10.5281/zenodo.8172728).

Various bottom‐dwelling species burrow or bury themselves into sand or mud, including some cephalopods (Hanlon & Messenger, 2018). For this taxon, it constitutes a cryptic behavior that aims to hide the individual from predators and/or prey (e.g., Boletzky, 1977). Burrowing implies movement through soft substrate (Dorgan, 2015) and has only been documented for octopuses (Hanlon & Messenger, 2018), which use their arms as shovels (Boletzky, 1996). On the other hand, burying, to cover with a thin layer of sediment (Dorgan, 2015), appears to be more widespread among cephalopods. Cuttlefish pump water from their funnel into the substrate and wait for the particles to settle upon them (fluidization), while bobtail “squids” move their arms at high frequency to gather sediment and completely cover themselves (fanning) (Boletzky, 1977; Mather, 1986). The surprising upside‐down positioning exhibited by the whiplash squid observed may allow it to continue pumping water over its gills via the siphon, which is held out of the mud. Although we cannot discern whether the individual had burrowed or buried itself, the cryptic mud‐covering behavior is evident and, to our knowledge, novel for true squid (i.e., orders Myopsida and Oegopsida) and even deep‐sea cephalopods.

While crypsis seeks to avoid the detection of an individual, masquerade acts against its recognition by self‐disguising as an object (Hanlon & Messenger, 2018). Here, the protuberant, still tentacles of the whiplash squid resembled, at least visually in artificial light from the ROV, biogenic structures commonly found in the region. These include the stalks of glass sponges, soft coral colonies or the tubes of large polychaete worms, all sessile, filter‐feeding animals frequently encountered in upper‐abyssal seascape communities at the Northeast Pacific (Simon‐Lledó et al., 2025; Simon‐Lledó, Bett, Huvenne, Schoening, Benoist, Jeffreys, et al., 2019). This masquerade could enhance the survival of the squid by concealing it from common cephalopod predators in the region, such as beaked whales (MacLeod et al., 2003) or rattail fish (*Coryphaenoides* sp.; Drazen et al., 2008). Even if masquerade is widespread among shallow‐water cephalopods (Hanlon et al., 2011), for squid, it had only been reported for the Caribbean reef squid *Sepioteuthis sepioidea*, which imitates floating algae (Hanlon & Messenger, 1996). Therefore, to the knowledge of the authors, this could be the first time that masquerade is observed for a deep‐sea cephalopod.

Another hypothesis is that the exposed tentacles displayed may be a form of aggressive mimicry, specifically Wicklerian–Eisnerian mimicry, when a predator disguises itself as an ally to its prey (Pasteur, 1982). Rigid biogenic structures like sponge stalks are biodiversity hotspots in the abyss that can host a wide range of epifauna and small crustaceans (Beaulieu, 2001), which are part of the diet of some *Mastigoteuthis* species (Kear, 1992). This would fit with the anatomy of *Mastigoteuthis* squids, which use their whip‐like tentacles, with numerous small suckers, as fly paper to capture slow‐moving prey on or above the sediment (Roper & Vecchione, 1997). This form of predation is possibly efficient in the abyss, where food is known to be scarce (Smith et al., 2008). Cephalopods show a decrease in metabolic rate with depth (Seibel et al., 1997), which is thought to explain why deep‐sea taxa use mainly fin swimming for locomotion instead of the more energy‐costly jet propulsion (Seibel et al., 2000). In contrast with burrowing/burying and masquerade, aggressive mimicry through tentacles has been documented in the deep sea, where bathypelagic squid *Grimalditeuthis bonplandi* undulates and flaps its clubs to attract prey (Hoving et al., 2013). Although we still know very little about the life habits of these taxa in the deep ocean (Hoving et al., 2014), this finding further expands the diversity of complex adaptations of cephalopods to life in the abyss.

Since the first seabed image surveys in the late 1970s, only four cephalopod taxa have been reported for the Northeast Pacific abyss, and whiplash squids had only been encountered once before (Simon‐Lledó et al., 2023b). In the Abyssal Pacific Seafloor Megafauna Atlas (Simon‐Lledó et al., 2023c), a regional catalogue of image‐identified invertebrates, these four cephalopod morphotypes are classified as Mastigoteuthidae gen. indet. (MOL_006; Figures 1 and 2b); *Cirroteuthis muelleri* sp. inc. (MOL_008; Figure 2d); *Grimpoteuthis* sp. indet. (MOL_010; Figure 2e); and Octopodidae gen. indet. (MOL_009; Figure 2c). Together, these taxa sum only 33 sightings across 5000 km of the CCZ (28 geographical locations and >150,000 m^2^ of surveyed seabed (Simon‐Lledó et al., 2023a); Figure 2a; Appendix S1: Table S1). As with the number of genetic species, the occurrences and distributions we report here are most certainly an underestimate, since (as this study shows) cephalopods can self‐disguise as well as sense and potentially avoid underwater sampling vehicles, which are the main source of our ecological and taxonomic knowledge for the abyssal Pacific.

This study unveils mud‐covering, masquerade, and aggressive mimicry behaviors that were previously undocumented for deep‐sea Cephalopoda. In turn, that Mastigoteuthidae squids may mimic animal stalks highlights the evolutionary importance of these biogenic structures as small‐scale biodiversity and animal abundance hotspots in the abyss (Beaulieu, 2001). Our study shows that, beyond their known role providing nesting sites (Purser et al., 2016), hard substratum structures like sponge stalks, in addition to polymetallic nodules (Simon‐Lledó, Bett, Huvenne, Schoening, Benoist, & Jones, 2019) or rocks (Mejía‐Saenz et al., 2023), appear to be of growingly high relevance to sustain the life habits of abyssal cephalopods, as shown in other groups. However, our discovery also demonstrates how little we still know about the diversity, distribution, and life history of abyssal taxa, particularly in the Northeast Pacific, where most of our knowledge solely stems from seabed imagery surveys. Moreover, the ability to self‐disguise helps explain the scarcity of squid sightings and suggests that current abundance and richness may be underestimated and that sedimentary structure could influence their distribution. Cephalopods play an important role in benthopelagic trophic food webs (Iken et al., 2001) by being one of the few groups in the abyss thought to be purely predatory and an important dietary component for peak predators (e.g., MacLeod et al., 2003). Thus, further investigations that specifically target cephalopods, for example, employing camera platforms (Robinson et al., 2021), are urgently required to protect the whole functional complexity and biodiversity of the abyssal Pacific seabed. This is timely as human impacts, such as ocean acidification from climate change (Harris et al., 2023) and seabed mining (Simon‐Lledó, Bett, Huvenne, Köser, Schoening, Greinert, & Jones, 2019), are expected to grow in these remote but vast ecosystems in the years to come.

## CONFLICT OF INTEREST STATEMENT

The authors declare no conflicts of interest.

## Supporting information


Appendix S1.

Video S1_Metadata.

Video S2_Metadata.

Video S1.

Video S2.

## Data Availability

Survey data and all cephalopod occurrences are available in Zenodo at https://doi.org/10.5281/zenodo.7982461 (Simon‐Lledó et al., 2023b).
