# Supplementary material for: Discovery of a mud‐covering cephalopod evidences the complex life habits in the abyss
Source: Ecology. 2025 Nov 25;106(11):e70257. doi: 10.1002/ecy.70257 (PMC12647930; doi:10.1002/ecy.70257)
Supplement: Supplementary file 1 — Appendix S1. [file ECY-106-e70257-s002.pdf]

Appendix S1

Discovery of a mud-covering cephalopod evidences the complex life habits in the abyss

Alejandra Mejía-Saenz, Bethany F.M. Fleming, Daniel O.B. Jones, Loïc Van Audenhaege, Henk-Jan Hoving, Erik Simon-Lledó

Journal: Ecology

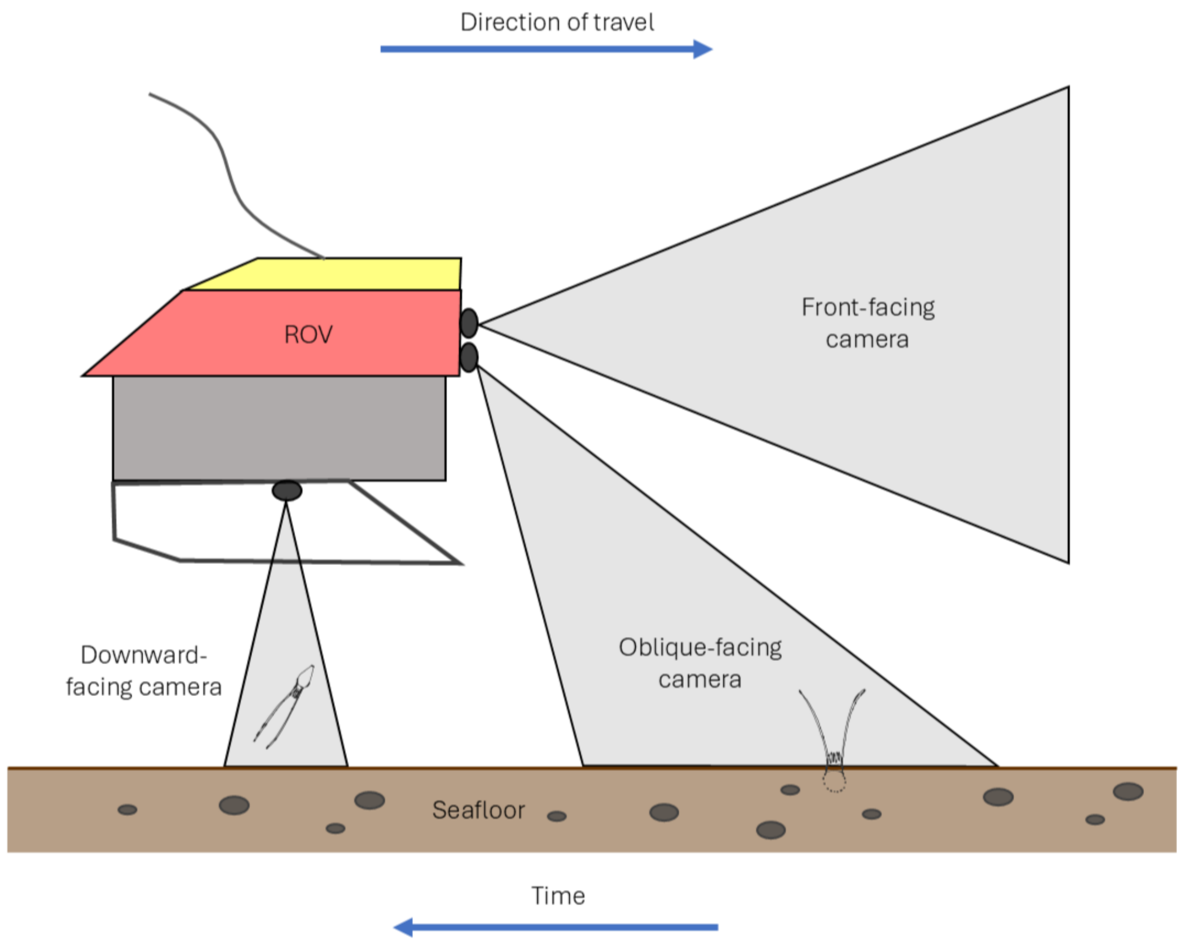

Figure S1. Simplified illustration of the remotely operated vehicle (ROV) *Isis* conducting a seafloor transect. The ROV is equipped with three cameras: one front-facing, one oblique-facing, and one downward-facing. It is connected to the research vessel via a cable and operated by a pilot, with guidance from a team of scientists onboard. As the ROV moves forward, specimens are typically seen first by the front- and oblique-facing cameras before entering the field of view of the downward-facing camera. In this transect, the squid was initially buried in sediment, visible in the oblique-facing camera, and was later observed swimming in the field of view of the downward-facing camera. For technical details about the ROV, cameras, and lighting setup, please refer to the cruise report (Jones and Glover 2023).

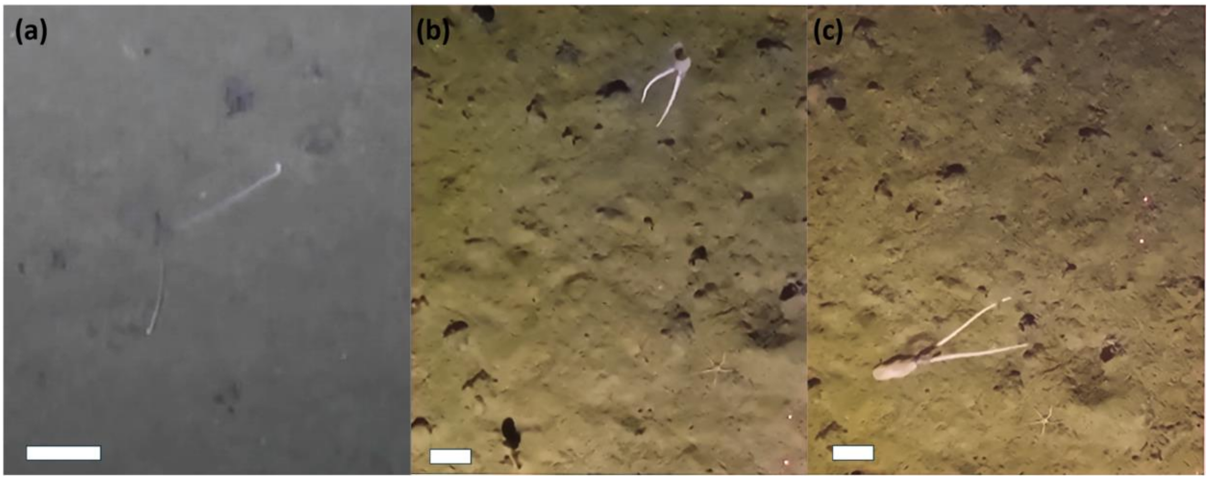

Figure S2. Image sequence depicting behaviour of specimen of whiplash squid Mastigoteuthidae gen. indet. (MOL_006 in Simon-Lledó et al. 2023b) using the ROV *Isis* during a seabed survey conducted at 4100 m depth in the abyssal northeast Pacific (Clarion-Clipperton Zone) on 17 March 2023 between 23:25:01 and 23:25:42 UTC. **a)** Specimen covered in soft sediment, motionless, with tentacles extended towards the water column, recorded in oblique facing footage. Extracted from Video S2; **b,c)** Recorded with downward-facing camera ~4 seconds after image 'a' and extracted from Video S1, specimen swimming **b)** upwards, **c)** perpendicular to the seafloor. Scale bars: 10 cm. Image credit: National Oceanography Centre / SMARTEX Project (NERC).

Table S1. Distribution of cephalopod taxa sighted at the Clarion-Clipperton Zone. Specimen described in the manuscript highlighted in bold. Taxa nomenclature based on Simon-Lledó et al. (2023b). Please refer to the corresponding source for details on the study areas and sampling methodologies. Lat = latitude; Long = longitude.

Taxon	Depth	Lat	Long	StudyArea	Zone	Source
MOL_008	5074	13.73662	-132.577	TOML_B	TMB_01	Simon-Lledó et al. 2023a
MOL_008	4964	10.04444	-131.781	KODOS	KIO_04	Simon-Lledó et al. 2023a
MOL_008	5008	6.993057	-149.912	APEI4	AP4_01	Simon-Lledó et al. 2023a
MOL_008	4937	9.883333	-131.733	KODOS	KIO_04	Simon-Lledó et al. 2023a
MOL_008	4937	9.883333	-131.733	KODOS	KIO_04	Simon-Lledó et al. 2023a
MOL_008	4809	13.77707	-132.644	TOML_B	TMB_01	Simon-Lledó et al. 2023a
MOL_008	4959	15.02629	-129.914	TOML_C	TMC_02	Simon-Lledó et al. 2023a
MOL_008	4887	9.980556	-131.617	KODOS	KIO_04	Simon-Lledó et al. 2023a
MOL_008	4921	13.65558	-132.604	TOML_B	TMB_02	Simon-Lledó et al. 2023a
MOL_008	4673	10.41889	-127.129	APEI-9	AP9_01	Simon-Lledó et al. 2023a
MOL_008	4930	13.78854	-132.615	TOML_B	TMB_01	Simon-Lledó et al. 2023a
MOL_008	4693	10.40972	-127.09	APEI-9	AP9_01	Simon-Lledó et al. 2023a
MOL_008	4210	11.234	-116.5	NORID	NORI_01	Simon-Lledó et al. 2023a
MOL_008	4900	13.764	-130.04	Nixo45	Nixo45	Tilot 2006
MOL_008	4900	13.764	-130.04	Nixo45	Nixo45	Tilot 2006
MOL_008	4900	13.764	-130.04	Nixo45	Nixo45	Tilot 2006
MOL_008	4900	13.764	-130.04	Nixo45	Nixo45	Tilot 2006
MOL_008	4470	14.56	-126.2	DomesC	DomesC	Foell and Pawson 1986
MOL_008	4005	19.55	-120.166	APEI-6 N	APEI -6 NE	Jones et al. 2021
MOL_008	4005	19.55	-120.166	APEI-6 N	APEI -6 NE	Jones et al. 2021
MOL_008	4005	19.55	-120.166	APEI-6 N	APEI -6 NE	Jones et al. 2021
MOL_008	4005	19.55	-120.166	APEI-6 N	APEI -6 NE	Jones et al. 2021

MOL_009	5048	10.50833	-131.336	KODOS	KIO_02	Simon-Lledó et al. 2023a
MOL_009	5048	10.50833	-131.336	KODOS	KIO_02	Simon-Lledó et al. 2023a
MOL_009	4186	11.00633	-116.143	NORID	NORI_01	Simon-Lledó et al. 2023a
MOL_009	4290	13.15563	-118.11	BGR	BGR	Simon-Lledó et al. 2023a
MOL_009	4594	13.85	-126.01	DomesC	DomesC	Foell and Pawson 1986
MOL_010	4004	19.12345	-120.543	APEI-6 N	APEI -6 NE	Jones et al. 2021
MOL_010	4010	18.8765	-120.123	APEI-6 N	APEI -6 NE	Jones et al. 2021
MOL_010	4293	12.85684	-118.401	BGR	BGR	Simon-Lledó et al. 2023a
**MOL_006**	**4099**	**13.95679**	**-116.531**	**UK-1**	**UK-1**	**Simon-Lledó et al. 2023a**
MOL_006	4090	17.28349	-122.875	APEI-6 S	AP6_01	Simon-Lledó et al. 2023a
MOL_008	4096	17.321	-122.053	APEI-6 S	AP6_02	Simon-Lledó et al. 2023a

References

Foell, Eric J., and David L. Pawson. 1986. “Photographs of Invertebrate Megafauna from Abyssal Depths of the North-Eastern Equatorial Pacific Ocean.” *The Ohio Journal of Science* 86:61–68.

Jones, Daniel O. B., and Adrian G. Glover. 2023. “Cruise Report: RRS James Cook Cruise JC241.” National Oceanography Centre. https://www.bodc.ac.uk/resources/inventories/cruise_inventory/reports/jc241.pdf.

Jones, Daniel O. B., Erik Simon-Lledó, Diva J. Amon, Brian J. Bett, Clémence Caulle, Louis Clément, Douglas P. Connelly, et al. 2021. “Environment, Ecology, and Potential Effectiveness of an Area Protected from Deep-Sea Mining (Clarion Clipperton Zone, Abyssal Pacific).” *Progress in Oceanography* 197 (September):102653. https://doi.org/10.1016/j.pocean.2021.102653.

Simon-Lledó, Erik, Diva J. Amon, Guadalupe Bribiesca-Contreras, Daphne Cuvelier, Jennifer M. Durden, Sofia P. Ramalho, Katja Uhlenkott, et al. 2023a. “Abyssal NE Pacific Seafloor Megafauna Dataset.” Zenodo. https://doi.org/10.5281/zenodo.7982462.

Simon-Lledó, Erik, Diva J. Amon, Guadalupe Bribiesca-Contreras, Daphne Cuvelier, Jennifer M. Durden, Sofia P. Ramalho, Katja Uhlenkott, et al. 2023b. “Abyssal Pacific Seafloor Megafauna Atlas,” March. https://zenodo.org/records/8172728.

Tilot, Virginie. 2006. “Biodiversity and Distribution of the Megafauna: Vol.1 The Polymetallic Nodule Ecosystem of the Eastern Equatorial Pacific Ocean, Vol.2 Annotated Photographic Atlas of the Echinoderms of the Clarion-Clipperton Fracture Zone, Vol. 3 Options for the Management and Conservation of the Nodule Ecosystem in Clarion Clipperton Fracture Zone.” 69. IOC Technical Series. UNESCO/IOC. https://unesdoc.unesco.org/ark:/48223/pf0000149556.
