# Supplementary material for: Discovery of a mud‐covering cephalopod evidences the complex life habits in the abyss
Source: Ecology. 2025 Nov 25;106(11):e70257. doi: 10.1002/ecy.70257 (PMC12647930; doi:10.1002/ecy.70257)
Supplement: Supplementary file 2 — Video S1_Metadata. [file ECY-106-e70257-s004.pdf]

Metadata for Video S1

Discovery of a mud-covering cephalopod evidences the complex life habits in the abyss

Alejandra Mejía-Saenz, Bethany F.M. Fleming, Daniel O.B. Jones, Loïc Van Audenhaege, Henk-Jan Hoving, Erik Simon-Lledó

Journal: Ecology

Video Caption

Video S1. Section of seabed survey using the downward-facing camera of ROV *Isis* conducted at 4100 m depth in the abyssal northeast Pacific (eastern Clarion-Clipperton Zone) from 23:25:21 to 23:25:42 on 17 March 2023 (UTC) during RRS James Cook Cruise JC241 (Dive 413 station JC241_098). Position: longitude -116.543 °, latitude 13.96706 ° (decimal degrees, WGS'84). The footage shows specimen of whiplash squid Mastigoteuthidae gen. indet. (MOL_006 in Simon-Lledó et al. 2023) swimming perpendicular to the seafloor. Recorded ~4 seconds after Video S2. For technical details about the ROV, cameras, and lighting setup, please refer to the cruise report (Jones and Glover 2023). Video credit: National Oceanography Centre and trustees of the Natural History Museum / SMARTEX Project (NERC).

ROV footage recorded by Antonio Calado; William Handley; Russell Locke; Stephen Mcdonagh; Emre Mutlu; Martin Yeomans; Bethany Fleming; Loïc Van Audenhaege; Erik Simon-Lledó; Guadalupe Bribiesca-Contreras; Adrian Glover; Daniel O. B. Jones.

References

Jones, Daniel O. B., and Adrian G. Glover. 2023. "Cruise Report: RRS James Cook Cruise JC241." National Oceanography Centre. https://www.bodc.ac.uk/resources/inventories/cruise_inventory/reports/jc241.pdf.

Simon-Lledó, Erik, Diva J. Amon, Guadalupe Bribiesca-Contreras, Daphne Cuvelier, Jennifer M. Durden, Sofia P. Ramalho, Katja Uhlenkott, et al. 2023. "Abyssal Pacific Seafloor Megafauna Atlas," March. https://zenodo.org/records/8172728.
