# Supplementary material for: Discovery of a mud‐covering cephalopod evidences the complex life habits in the abyss
Source: Ecology. 2025 Nov 25;106(11):e70257. doi: 10.1002/ecy.70257 (PMC12647930; doi:10.1002/ecy.70257)
Supplement: Supplementary file 3 — Video S2_Metadata. [file ECY-106-e70257-s005.pdf]

Metadata for Video S2

Discovery of a mud-covering cephalopod evidences the complex life habits in the abyss

Alejandra Mejía-Saenz, Bethany F.M. Fleming, Daniel O.B. Jones, Loïc Van Audenhaege, Henk-Jan Hoving, Erik Simon-Lledó

Journal: Ecology

Video Caption

Video S2. Section of seabed survey using the oblique-facing camera *Scorpio* of ROV *Isis* conducted at 4100 m depth in the abyssal northeast Pacific (eastern Clarion-Clipperton Zone) from 23:25:01 to 23:25:17 on 17 March 2023 (UTC) during RRS James Cook Cruise JC241 (Dive 413 station JC241_098). Position: longitude -116.543 °, latitude 13.96707 ° (decimal degrees, WGS'84). The footage shows specimen of whiplash squid Mastigoteuthidae gen. indet. (MOL_006 in Simon-Lledó et al. 2023) covered in soft sediment, motionless, with tentacles extended towards the water column. Recorded ~4 seconds before Video S1. Red circle overlaid to aid visualisation. For technical details about the ROV, cameras, and lighting setup, please refer to the cruise report (Jones and Glover 2023). Video credit: National Oceanography Centre and trustees of the Natural History Museum / SMARTEX Project (NERC).

ROV footage recorded by Antonio Calado; William Handley; Russell Locke; Stephen Mcdonagh; Emre Mutlu; Martin Yeomans; Bethany Fleming; Loïc Van Audenhaege; Erik Simon-Lledó; Guadalupe Bribiesca-Contreras; Adrian Glover; Daniel O. B. Jones.

References

Jones, Daniel O. B., and Adrian G. Glover. 2023. "Cruise Report: RRS James Cook Cruise JC241." National Oceanography Centre. https://www.bodc.ac.uk/resources/inventories/cruise_inventory/reports/jc241.pdf.

Simon-Lledó, Erik, Diva J. Amon, Guadalupe Bribiesca-Contreras, Daphne Cuvelier, Jennifer M. Durden, Sofia P. Ramalho, Katja Uhlenkott, et al. 2023. "Abyssal Pacific Seafloor Megafauna Atlas," March. https://zenodo.org/records/8172728.
